# To what extent does IQ 'explain' socio-economic variations in function?

**DOI:** 10.1186/1471-2458-7-179

**Published:** 2007-07-25

**Authors:** Hans Bosma, Martin PJ van Boxtel, Gertrudis IJM Kempen, Jacques ThM van Eijk, Jelle Jolles

**Affiliations:** 1 Maastricht University, Social Medicine, PO Box 616, 6200 MD Maastricht, The Netherlands; 2 Maastricht University, Psychiatry and Neuropsychology, PO Box 616, 6200 MD Maastricht, The Netherlands; 3 Maastricht University, School for Public Health and Primary Care, P.O. Box 616, 6200 MD Maastricht, The Netherlands

## Abstract

**Background:**

The aims of this study were to examine the extent to which higher intellectual abilities protect higher socio-economic groups from functional decline and to examine whether the contribution of intellectual abilities is independent of childhood deprivation and low birth weight and other socio-economic and developmental factors in early life.

**Methods:**

The Maastricht Aging Study (MAAS) is a prospective cohort study based upon participants in a registration network of general practices in The Netherlands. Information was available on 1211 men and women, 24 – 81 years old, who were without cognitive impairment at baseline (1993 – 1995), who ever had a paid job, and who participated in the six-year follow-up. Main outcomes were longitudinal decline in important components of quality of life and successful aging, i.e., self-reported physical, affective, and cognitive functioning.

**Results:**

Persons with a low occupational level at baseline showed more functional decline than persons with a high occupational level. Socio-economic and developmental factors from early life hardly contributed to the adult socio-economic differences in functional decline. Intellectual abilities, however, took into account more than one third of the association between adult socio-economic status and functional decline. The contribution of the intellectual abilities was independent of the early life factors.

**Conclusion:**

Rather than developmental and socio-economic characteristics of early life, the findings substantiate the importance of intellectual abilities for functional decline and their contribution – as potential, but neglected confounders – to socio-economic differences in functioning, successful aging, and quality of life. The higher intellectual abilities in the higher socio-economic status groups may also underlie the higher prevalences of mastery, self-efficacy and efficient coping styles in these groups.

## Background

Lately there have been reports that lower intellectual abilities may result in poor physical functioning and even in heightened risks of mortality [1-9]. The mechanisms underlying the association, however, remain undetermined. In particular, the issue of whether lower intellectual abilities are related to poor health outcomes, independent of adverse socio-economic conditions in childhood and adulthood, remains unresolved [5,10]. A related unresolved issue is whether the association between low socio-economic status and poor health is confounded by lower intellectual abilities in the lower socio-economic groups [11,12]. In this context, intelligence has even been postulated as the "elusive fundamental cause of social class inequalities in health" [13]. Given strong associations between intellectual abilities and where people end up in the socio-economic hierarchy [14], taking into account potential confounding by intellectual abilities perhaps should indeed have an increased priority in studies of socio-economic differences in health. A recent finding of the London-based Whitehall II study showed that intelligence probably is not the driving force behind socioeconomic inequalities in health in white-collar workers [15]. Another recent study, however, showed that controlling for intelligence led to a marked reduction in the magnitude of the socioeconomic gradients in health [16]. In both studies, health measures varied from self-reported mental and physical functioning to coronary heart disease and all-cause mortality.

**Figure 1:**
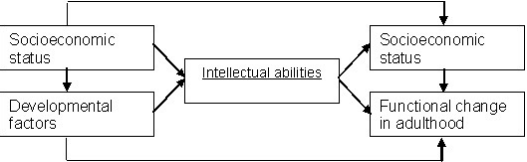
The research model.

Using longitudinal data from the Maastricht Aging Study (MAAS), the present study further examines whether the higher intellectual abilities in the high socio-economic status groups protect these groups from declines in reports of physical, affective and cognitive functioning. It is of relevance to study individual perceptions of physical, affective, and cognitive functioning, because these have been identified as valid indicators of quality of life [17] and successful aging [18]. The person-centered (rather than doctor-centered) perspective is increasingly acknowledged an equivalent outcome measure. Increasingly, these measures are also used in clinical trials, as the personal experience might be more decisive for using health care services than many so-called "objective" measures [17]. Given that intellectual abilities may have been negatively influenced by adverse socioeconomic conditions in childhood (e.g. deprivation) [19] or adverse developmental factors (e.g. low birth weight, childhood disease) [20], these characteristics were taken into account. Figure 1 further clarifies the underlying research model.

## Methods

### Study population

MAAS started as a prospective study of the determinants of normal cognitive aging [21,22]. Participants in MAAS were recruited from a registration network of general practices in the South of The Netherlands. Twenty-four to eighty-one year old men and women were considered eligible. Individuals were excluded if they had a history of stroke, mental retardation, or chronic neurological pathology (e.g. dementia, epilepsy, Parkinson, or central nervous system malignancy). Participants were stratified by age (12 groups), sex, and occupational achievement (two groups). At baseline (1993 – 1995), 1821 individuals were medically and neuropsychologically tested at the University laboratory. A questionnaire had to be filled out at home. Six years later (1999 – 2001), 1376 (75.6%) individuals returned for follow-up assessment. A group of 271 (14.9%) individuals refused further participation, 118 (6.3%) had died, 37 (2.0%) were medically unfit to participate and 19 (1.0%) did not take part for other reasons. MAAS was approved by the Medical Ethics Committee of the University Hospital Maastricht. All participants gave their written informed consent.

### Physical, affective, and cognitive functioning

At both baseline and follow-up phase, physical functioning was determined by asking whether or not people – due to their physical condition – needed help with the following activities (Instrumental Activities of Daily Living): shopping, household chores, preparation of meals, personal care, and getting dressed (internal consistency measure: Cronbach's α = 0.72). These items were summed. Affective functioning was determined by the depression subscale of the widely used Symptom Checklist [23]. There are 16 five-category items asking whether persons were hindered by depressive thoughts and feelings during the last week (Cronbach's α = 0.89). The items were summed. Cognitive functioning was determined by a question asking about bother due to forgetfulness in daily life (1: not at all – 5: very much). This information has been shown to be related to health care consumption for cognitive complaints and has been used as an indicator of cognitive capacity in several large-scale studies (e.g. [24-26]). All three measures were subsequently rescaled into variables ranging from 0 (good function) to 100 (poor function).

### Adult socio-economic status

Socio-economic status was defined by the respondents' occupational level using the International Socio-Economic Index of Occupational Status (ISEI) based upon job title codes, as used by Statistics Netherlands [27]. The resulting score was divided into thirds (using tertiles). There were 165 persons who never had any paid job.

### Intellectual abilities

At baseline, intellectual abilities were measured with the abbreviated version of the Groningen Intelligence Test (GIT) [28]. The GIT is the most frequently used intelligence test of formal IQ in The Netherlands and, although analogous to the Wechsler Adult Intelligence Scale, relies less on the persons' verbal abilities. There are five sub-tests: analogies, fluency, doing sums, vocabulary, and mental rotation. The sub-tests correlations varied from 0.09 (between mental rotation and doing sums) to 0.54 (between mental rotation and analogies). One composite score reflecting the full scale intelligence level was created (sample mean = 115.4; sd = 13.1).

### Early life socioeconomic conditions

Socioeconomic conditions during early life were measured with the educational level of the father and mother (both seven categories), and the occupational level of the father (ISEI index) [27]. These variables were divided into thirds (using tertiles). Deprivation in childhood was measured with a question about whether, in childhood, there was frequently not enough money to buy basic goods (e.g. food, clothes).

### Early life developmental factors

Early developmental factors were measured with retrospective reports about whether or not persons as a child or baby experienced an assisted birth, low birth weight (less than 2500 grams), nuchal cord (i.e. umbilical cord wrapped around baby's neck), or delayed developmental milestones (e.g. late start talking, walking). Severe diseases during childhood or adolescence were measured by asking whether or not respondents had to repeat classes at school due to severe illnesses.

### Statistical analysis

Firstly, basic statistics (percentages and means) of all covariates are compared between occupational groups. Secondly, associations between socio-economic status and physical, affective, and cognitive functioning at baseline and at the follow-up phase and the six-year changes were analyzed with analysis of variance, controlling for age and sex (and for baseline functioning, when analyzing the association with changes in functioning). Thirdly, associations of early socioeconomic and developmental factors and intellectual abilities with six-year changes in physical, affective, and cognitive functioning were examined by linear regression analyses, controlling for age, sex, and baseline functioning. Fourthly, the association between adult occupational level and changes in functioning was analyzed with linear regression analyses. Three models were estimated and compared: model 1 was adjusted for age, sex, and baseline functioning; model 2 was additionally adjusted for early socioeconomic and developmental factors; and model 3 was additionally adjusted for intellectual abilities. This hierarchy of models quantifies the contribution of intellectual abilities (to the socio-economic differences in function), net of (i.e. corrected for) the potentially confounding influence of developmental and socioeconomic factors from childhood. The percent decrease of the unstandardised regression coefficients for occupational level, when including new variables, indicates the extent to which the new variables 'explain' the socioeconomic effects. To increase power, these latter analyses were also done with the continuous measures of both occupational level and intellectual abilities. Assumptions of linear regression were checked and found to be not violated.

## Results

Table 1 shows that there were fewer women in the higher occupational groups (35 versus 42% in the lower occupational groups). Intellectual abilities were also lower in these groups; only 10 percent had lower intellectual abilities compared with 49 percent in the lower occupational groups. Current occupational level was also strongly related to the socioeconomic conditions in childhood. Of the early developmental factors (all with rather low prevalences), only delayed milestones was significantly less common in the higher occupational groups.

**Table 1:** Characteristics of the study population by occupational level

		Occupational level:		
	
	Total	High	Intermediate	Low
	(n = 1211)	(n = 385)	(n = 422)	(n = 404)
Women (%)	48.6	34.5	57.3	41.8 *
Age, mean (SD)	50.3 (15.4)	50.2 (14.7)	48.5 (15.3)	50.1 (15.1)
Low intellectual abilities (%)	31.6	10.4	27.7	48.8 *
*Early socioeconomic conditions*				
Deprivation (%)	7.0	4.8	5.7	12.7 *
Low occupational level of father (%)	36.0	26.6	33.4	47.0 *
Low educational level of father (%)	54.9	44.7	53.7	63.5 *
Low educational level of mother (%)	65.5	59.0	60.3	73.9 *
*Early developmental factors*				
Assisted birth (%)	5.0	5.8	3.9	5.6
Low birth weight (%)	4.4	3.0	5.2	5.4
Nuchal cord (%)	2.4	2.3	1.9	3.4
Delayed milestones (%)	4.6	2.4	6.5	4.3 *
Severe disease in childhood (%)	7.2	4.9	7.6	9.2

* *p *< .05.

There was no association between adult occupational level and physical, affective, and cognitive functioning at baseline (Table 2). Except for men and women with a high occupational level who experienced positive changes in their cognitive functioning (-0.91), all groups showed declines in all three types of functioning (reflected in the positive change scores). The strongest declines occurred in the lowest socioeconomic group. Given the theoretical range of the functioning measures (from 0: good to 100: poor), average scores, longitudinal declines, and socioeconomic differences therein were small.

**Table 2:** Mean physical, affective, and cognitive functioning at baseline and six-year follow-up phase, according to adult occupational level, adjusted for age and sex (analysis of variance); mean six-year functional change score is additionally adjusted for baseline functioning (analysis of variance) (*N *= 1211)^a^

	Mean functioning:		
	At baseline	At follow-up	Six-year change

Physical functioning			
High occupational level	2.97	4.35	1.87
Intermediate level	2.05	4.24	2.31
Low occupational level	2.30	6.76 *	4.29 *
Affective functioning			
High occupational level	6.83	15.95	9.12
Intermediate level	6.75	16.56	9.85
Low occupational level	6.93	18.79 *	11.94 *
Cognitive functioning			
High occupational level	17.39	16.40	-0.91
Intermediate level	15.73	16.83	0.46
Low occupational level	18.35	20.53 *	3.05 *

* *p *< .05.

^a ^Measures for functioning range from 0 (good) to 100 (poor); change score indicates follow-up minus baseline score. Positive values for the change score indicate longitudinal decline; negative values indicate improvement.

Intellectual abilities were lower in persons with poor socioeconomic conditions during childhood or adulthood (not tabulated). Those with reports of severe diseases in childhood also had lower intellectual abilities. Other developmental factors were not related to the intellectual abilities.

Table 3 shows that early socioeconomic conditions were not consistently related to the three different measures of functional decline. Childhood deprivation, father's low educational level, and father's occupational level were significantly related to adverse physical, affective, and cognitive changes, respectively. The coefficient of 3.94 indicates that reports of childhood deprivation were related to 3.94 points more six-year decline in physical functioning compared with better-off counterparts. Early developmental factors were not related to functional decline, except for a strong adverse effect of delayed milestones on declining physical function (b coefficient: 4.90). Low intellectual abilities, on the other hand, were strongly related to declines across all three types of functional change.

**Table 3:** Regression coefficients (b) for socioeconomic and developmental conditions during childhood and intellectual abilities predicting six-year change in physical, affective, and cognitive functioning in adulthood, adjusted for age, sex, and baseline level of functioning (*N *= 1211)^a^

		Six-year change in:					
		Physical functioning		Affective functioning		Cognitive functioning	
		
		B	95% CI	B	95% CI	B	95% CI

*Early socioeconomic conditions*							
Deprivation	No	0	(reference)	0	(reference)	0	(reference)
	Yes	3.94	(1.26, 6.61)	1.66	(-1.44, 4.77)	-0.09	(-3.89, 3.71)
Occupational level of father	High	0	(reference)	0	(reference)	0	(reference)
	Intermediate	-0.15	(-1.96, 1.65)	0.85	(-1.24, 2.95)	2.51	(0.02, 5.01)
	Low	-0.08	(-1.78, 1.61)	1.54	(-0.41, 3.50)	3.72	(1.40, 6.05)
Educational level of father	High	0	(reference)	0	(reference)	0	(reference)
	Intermediate	-0.42	(-2.48, 1.65)	3.29	(0.75, 5.82)	2.57	(-0.52, 5.67)
	Low	0.27	(-1.51, 2.04)	2.86	(0.70, 5.02)	2.96	(0.32, 5.60)
Educational level of mother	High	0	(reference)	0	(reference)	0	(reference)
	Intermediate	0.44	(-1.94, 2.82)	-0.22	(-3.09, 2.65)	1.15	(-2.36, 4.65)
	Low	-0.36	(-2.46, 1.75)	0.91	(-1.63, 3.44)	0.61	(-2.47, 3.70)
*Early developmental factors*							
Assisted birth	No	0	(reference)	0	(reference)	0	(reference)
	Yes	0.68	(-2.75, 4.11)	-1.77	(-5.54, 2.00)	0.03	(-4.71, 4.76)
Low birth weight	No	0	(reference)	0	(reference)	0	(reference)
	Yes	1.24	(-2.03, 4.51)	-0.47	(-4.47, 3.53)	-1.04	(-5.86, 3.77)
Nuchal cord	No	0	(reference)	0	(reference)	0	(reference)
	Yes	-0.09	(-4.46, 4.27)	1.57	(-3.83, 6.98)	0.53	(-0.72, 1.78)
Delayed milestones	No	0	(reference)	0	(reference)	0	(reference)
	Yes	4.90	(1.65, 8.14)	0.07	(-3.94, 4.07)	0.68	(-4.35, 5.71)
Severe disease in childhood	No	0	(reference)	0	(reference)	0	(reference)
	Yes	0.16	(-2.47, 2.79)	1.41	(-1.63, 4.45)	1.27	(-2.39, 4.94)
*Intellectual abilities*	High	0	(reference)	0	(reference)	0	(reference)
	Intermediate	1.83	((0.14, 3.52)	0.44	(-1.51, 2.38)	-0.33	(-2.68, 2.02)
	Low	2.92	(1.16, 4.69)	2.37	(0.33, 4.41)	4.60	(2.14, 7.06)

^a ^Reference category is 'high' for occupational level of the father, educational level of the parents, and intellectual abilities. Reference category is 'no' for all other variables.

Compared with persons with a high occupational level, persons with a low occupational level declined 2.43, 2.81 and 3.96 points more during the six-year follow-up compared with persons with a high occupational level (Table 4). An early socioeconomic indicator was created by counting the number of times persons were in the poorest category across the four early socioeconomic variables. An early developmental indicator was similarly created from the developmental items. When introduced in model 2, the coefficients for socioeconomic status did not change substantially. The socioeconomic coefficients for changes in affective functioning decreased with 19 and 11%, respectively, indicating that a small part of the socioeconomic differences in affective functioning may be based on early life factors. When the intellectual abilities were (additionally) introduced in model 3, the coefficients of socioeconomic status decreased substantially. More than one third of the association between adult occupational level and longitudinal changes in physical, affective, and cognitive functioning was taken into account by the lower intellectual abilities in the lower occupational groups. In the latter model, none of the coefficients related to socioeconomic status remained statistically significant.

**Table 4:** Regression coefficients (b) for adult occupational level predicting six-year change in physical, affective, and cognitive functioning in adulthood. adjusted for age, sex, and baseline level of functioning (Model 1), additionally adjusted for childhood socioeconomic conditions and developmental factors in early life (Model 2), and additionally adjusted for intellectual abilities (Model 3) (*N *= 1211)^a^

	Model 1		Model 2			Model 3		
	
	B	95% CI	B	95% CI		B	95% CI	
Physical functioning								
High occupational level	0.00	(reference)	0.00	(reference)		0.00	(reference)	
Intermediate level	0.44	(-1.29, 2.17)	0.41	(-1.33, 2.15)	[07]	-0.12	(-1.88, 1.63)	[>100]
Low occupational level	2.43	(0.70, 4.15)	2.44	(0.68, 4.20)	[00]	1.02	(-0.89, 2.94)	[58]
*Continuous measures *^*b*^	*0.07*	*(0.02, 0.12)*	*0.07*	*(0.02, 0.12)*	*[00]*	*0.03*	*(-0.03, 0.08)*	*[57]*
Affective functioning								
High occupational level	0.00	(reference)	0.00	(reference)		0.00	(reference)	
Intermediate level	0.73	(-1.32, 2.78)	0.59	(-1.47, 2.65)	[19]	0.26	(-1.83, 2.36)	[56]
Low occupational level	2.81	(0.77, 4.86)	2.49	(0.40, 4.58)	[11]	1.73	(-0.56, 4.01)	[31]
*Continuous measures*	*0.08*	*(0.02, 0.13)*	*0.07*	*(0.01, 0.12)*	*[13]*	*0.04*	*(-0.02, 0.11)*	*[50]*
Cognitive functioning								
High occupational level	0.00	(reference)	0.00	(reference)		0.00	(reference)	
Intermediate level	1.37	(-1.13, 3.86)	1.31	(-1.20, 3.81)	[04]	0.70	(-1.85, 3.24)	[47]
Low occupational level	3.96	(1.47, 6.44)	3.84	(1.30, 6.38)	[03]	2.44	(-0.33, 5.22)	[37]
*Continuous measures*	*0.12*	*(0.05, 0.19)*	*0.12*	*(0.05, 0.19)*	*[00]*	*0.08*	*(0.00, 0.16)*	*[33]*

^a ^Between brackets is the percentage decline of the B-coefficient compared with the previous model. It indicates the extent to which newly introduced variables 'explain' the association of adult occupational level with physical, affective, and cognitive functioning. ^*b *^Analyses done using continuous measures of both intellectual abilities and occupational level (post-recode range: -87 (high) to -16 (low)).

Findings were even more pronounced when the continuous measures of both occupational level and intellectual abilities were used (Table 4). On average, early life factors hardly contributed to the socioeconomic differences in functional decline, while intellectual abilities even had a higher contribution. Of the socioeconomic differences in physical, affective, and cognitive decline, 57, 50, and 33 percent, respectively, were taken into account by intellectual abilities.

Table 5 presents the findings for the five sub-tests of the GIT intelligence test. It shows that the vocabulary sub-test took into account most of the effect of occupational level. At least 37 percent of the adverse effect of low adult occupational level on functional decline was taken into account by lower vocabulary scores in lower occupational groups. The highest contribution of the vocabulary sub-test holds across all three domains of functional decline. The effect of occupational level was not significant in any model with vocabulary scores included (not tabulated).

**Table 5:** Percentage of the effect of low and intermediate level adult occupation (compared with high occupational level) taken into account by sub-tests of the Groningen Intelligence Test ^a^

	% taken into account by:				
	
	Doing Sums	Vocabulary	Mental Rotation	Analogies	Fluency
Physical functioning					
High occupational level	(reference)	(reference)	(reference)	(reference)	(reference)
Intermediate level	49 %	>100 %	22 %	100 %	34 %
Low occupational level	44 %	64 %	07 %	34 %	14 %
Affective functioning					
High occupational level	(reference)	(reference)	(reference)	(reference)	(reference)
Intermediate level	09 %	59 %	34 %	66 %	00 %
Low occupational level	15 %	37 %	12 %	28 %	00 %
Cognitive functioning					
High occupational level	(reference)	(reference)	(reference)	(reference)	(reference)
Intermediate level	21 %	45 %	05 %	30 %	24 %
Low occupational level	23 %	37 %	02 %	16 %	18 %

^a ^The percentage is the percentage decline of the B-coefficients of the effect of adult occupational level in the model with the particular intelligence sub-test included compared with the model without that sub-test (i.e. model 2 in Table 3).

Intellectual abilities had similar effects in low and high socio-economic status groups and in the young and old (not tabulated). The contribution of the intellectual abilities to the socio-economic differences in functioning was somewhat stronger in men than in women. Furthermore, associations were similar using educational and income level as indicators of socioeconomic status (not tabulated). The associations with income level were somewhat weaker though, perhaps because income was only measured at the follow-up phase. The analyses with the alternative socioeconomic measures included the 165 persons who never had any paid job and who were thus excluded in the analyses with occupational level. In contrast to the occupational level indicator though, educational and income level were also related to baseline affective and cognitive functioning.

## Discussion

Lower intellectual abilities in the lower socio-economic status groups took into account more than one third of the reported socio-economic differences in the decline in important components of quality of life and successful aging, i.e physical, affective, and cognitive functioning. None of the socioeconomic differences in functional decline remained statistically significant, when intellectual abilities were included in the model. The contribution of intellectual abilities was independent of the potentially confounding influence of early life socioeconomic conditions, including childhood deprivation, and early life developmental factors, including low birth weight. This was particularly due to only small effects of these early conditions on later functional decline.

A major drawback of our study is that the intellectual abilities were measured at baseline, when the respondents were 25 years old or older. We cannot exclude the possibility that intellectual abilities have changed as a result of socioeconomic circumstances and related work characteristics (rather than vice versa). The vocabulary sub-test of the GIT contributed most strongly to the association between socio-economic status and longitudinal decline in functioning. This is relevant here, as such crystallized abilities are thought to be particularly sensitive to educational experiences [29]. But, also if intellectual abilities can be stimulated by socioeconomic circumstances, such as being in an active job [30] or having a high education, our findings emphasize the importance of intellectual abilities for socioeconomic differences in health and functioning. The study of Batty and colleagues also reported substantial "explanation" by differences in intelligence [16]. Recent Whitehall II findings, on the contrary, indicated that intellectual abilities are probably not the driving force behind socioeconomic differences in health [15]. It is unclear how to explain this contrast among findings, but differences in the study population's composition, research design, age range, and measures of socioeconomic grading, health status, and intellectual abilities may be important. Foremost, our findings indicate that low socio-economic status cannot be established as a risk factor or indicator for functional decline, poor quality of life, and unsuccessful aging, until the possibility of confounding by intellectual abilities is fully excluded [11,13].

Our findings thus show that intelligence is also informative for future deterioration of experienced quality of life. The personal functional experience and its "objective" counterparts, such as physical, performance-based tests or cognitive, neuropsychological tests are not necessarily perfectly related [31]. Equally well, there is no perfect relation between the reported functional decline and disease [31]. As mentioned previously, however, such quality of life measures are important for their patient-centeredness and their relevance for use of health care services [17,18]. Moreover, the findings may shed some further light on the complexity of the mechanisms linking intelligence and premature mortality (as reported by others, e.g. [16]). As we will discuss below, personal perceptions of coping and mastery – related to functional outcomes – may be important here (see also [32]). As there were only few incident cases of coronary heart disease and all-cause mortality, these outcomes must await examination after longer follow-up intervals. In our data, prevalent disease appeared not to contribute to socioeconomic differences in functioning (see below) [33]. In previous studies, however, findings were about similar across health measures, including (coronary heart disease) mortality and self-reports of physical and mental functioning [15,16]. However, the IQ contribution was somewhat stronger for the mortality outcomes in Batty and colleagues' study [16].

Lower intellectual abilities might affect rates of decline in functioning through adverse health behaviours [9,34-37]. Unawareness of the consequences of unhealthy behaviours, such as smoking or a sedentary lifestyle, may be a mediating pathway. A related mediating pathway in the association between intellectual abilities and functioning might be via somatic diseases [4,6,8]. Perhaps persons with lower abilities have higher prevalences of disease (via their unhealthy behaviours might be one route). However, we have shown that prevalent adult diseases and health behaviours (and life-events) hardly contributed to the socioeconomic differences in physical, affective, and cognitive functioning and thus that intellectual abilities were probably not related to declines in functioning through behavioural or disease-related pathways [33]. Although the present study is not about mechanisms underlying the association between intellectual abilities and declines in functioning in varying domains, it is striking to find in our data a positive association between intellectual abilities and a measure of control beliefs (mastery) (these beliefs were measured at the follow-up phase only). The Pearson correlation was 0.26 (p = 0.05) (not tabulated). This suggests that higher intellectual abilities may help people to cope more easily with daily hassles, life events, and chronic stressful circumstances. Batty and Deary also postulated the possibility of a route via stress management skills as one of the mechanisms through which intelligence affects health [34]. Intelligence is about effectively dealing with complexity. Effective experiences, particularly in adverse and complex circumstances, are likely to increase levels of mastery and self-efficacy. Having to adhere to complex treatment regimens may be one such stressful circumstance with which the higher classes – because of their higher intellectual abilities – cope more effectively [6,11,34].

### Further methodological considerations

Some further methodological issues should be discussed. Firstly, the psychometric quality of the outcome measures could be questioned. The physical functioning items showed a moderate to high internal consistency (Cronbach's α = 0.72) and strongly resemble items in well-known scales of instrumental activities of daily living [38]. The affective functioning items, though restricted to depression, come from a depression scale that has a high reliability and validity (Cronbach's α in our study = 0.89) [23]. The cognitive function item asks for bother due to forgetfulness. Such complaint is not necessarily strongly related to test performance. A recent study, however, found that persons with cognitive complaints (but normal test performance) showed brain atrophy similar to that of amnestic mild cognitive impairment [39]. Another recent study found that perceived memory function was a predictor of subsequent memory performance [40]. Other research confirms the importance of self-reports [24-26,41]. Important here is also that intelligence is related to changes in the reported functioning (controlling for the baseline score) which indicates that reporting bias (e.g. negative affectivity) is less likely; negative affectivity would similarly bias reports of both baseline and follow-up reports of functioning and its bias is avoided when analyzing change.

Secondly, not many experienced major declines in functioning and most started from high levels of functioning. Residuals in the linear regression were, however, normally distributed, and findings were similar in old persons where there is more poor function and longitudinal decline. The high mean level of intellectual abilities (mean = 115.4; SD = 13.1) may also be indicative of the initially well-functioning MAAS cohort. Thirdly, persons with a low socioeconomic status, as well as persons with poor functioning and persons with low intellectual abilities more often dropped out during the study (particularly due to refusal rather than death or any other cause of attrition) [42]. This pattern of attrition may underlie the small six-year functional decline (from a high level of functioning) and the small socio-economic status and intelligence-related differences therein. It cannot, however, be determined how this pattern might have affected our finding of a substantial contribution of intellectual abilities. Fourthly, persons with a mental retardation were excluded from MAAS at baseline. Furthermore, excluding outliers and influential cases did not result in different findings. Hence, findings are probably not based on few persons with extreme low scores on the intellectual abilities measure.

Fifthly, birth weight and complications during birth and other developmental factors were based on self-reports and may therefore have been subject to recall bias, especially in older persons [43,44]. However, the analysis of functional decline rather than momentary function excluded the possibility that overreports of adversities in childhood by those with poor functional outcomes could cause overestimated associations between childhood factors and our functional outcome. The extent of non-differential reporting bias is still unclear, as is the extent to which this might underlie the absence of effects of these factors on decline in adulthood.

Sixthly, if our measure of intellectual abilities also picks up characteristics, such as verbal abilities and differences in being used to test-taking, higher occupational level groups might have (artificially) higher scores on the particular measure. More research is needed to examine this issue in more detail [45]. Seventhly, the educational level of the parents might be a surrogate measure of the parents' intellectual ability levels, through which any effect of the parents' education might actually be confounded. In the absence of a consistent effect of the parents' education, such confounding has not likely played a major role. Finally, findings should be interpreted cautiously, because it is unclear why education and income, but not occupation, were related to affective and cognitive function at baseline.

## Conclusion

Rather than developmental (e.g. birth weight) and socio-economic (e.g. deprivation) characteristics of early life, our findings substantiate the importance of intellectual abilities for physical, affective, and cognitive decline and their contribution – as potential, but neglected confounders – to socio-economic differences in functioning, successful aging, and quality of life. The higher intellectual abilities in the higher socio-economic status groups may also underlie the higher prevalences of mastery, self-efficacy and efficient coping styles in these groups.

## Competing interests

The author(s) declare that they have no competing interests.

## Authors' contributions

HB formulated the research questions, analysed the data, and wrote the paper. MPJvB and JJ coordinated data collection, contributed to the interpretation of the data and critically read the manuscript. GIJMK and JThMvE contributed to the interpretation of the data and critically read the manuscript. All authors approved the final manuscript.

## Pre-publication history

The pre-publication history for this paper can be accessed here:


